# Supplementary material for: Screening attendance of breast or cervical cancers and its associated factors among 30–49 year old women in Gedeo zone, South Ethiopia: Cross-sectional study
Source: PLoS One. 2025 Jan 31;20(1):e0315891. doi: 10.1371/journal.pone.0315891 (PMC11785269; doi:10.1371/journal.pone.0315891)
Supplement: Attachment — Submitted filename: Response to Reviewers.docx [file pone.0315891.s001.docx]

**Editor comment**

1. **Comment:** Please consider all reviewers' comments. Particularly, those regarding typos and English improvements. For example, in tables with missing information, you can describe these frequencies as "No Available-NA" instead of "Don't know".

**Response:** Thank you for your valuable feedback and the insightful comments from the reviewers. We appreciate the time and effort dedicated to reviewing our manuscript. We have carefully considered all the comments, especially those regarding typos and improvements in English usage. Regarding the tables where "Don't know" is listed as a response option, we have clarified that this is one of the available responses (along with "Yes" and "No"). For additional clarity, we have uploaded the questionnaire as a supplementary file.

2. **Comment:** We note that you have indicated that there are restrictions to data sharing for this study. PLOS only allows data to be available upon request if there are legal or ethical restrictions on sharing data publicly.

**Response:** Thank you for your insight regarding data availability. We would like to clarify that all data files for this study are publicly accessible through the City of Hope National Medical Center database. The link to access these files is provided in the data availability statement included in our online submission.

3. **Comment:** Please include your full ethics statement in the ‘Methods’ section of your manuscript file. In your statement, please include the full name of the IRB or ethics committee who approved or waived your study, as well as whether or not you obtained informed written or verbal consent. If consent was waived for your study, please include this information in your statement as well.

**Response:** Thank you for your guidance regarding the ethics statement. We appreciate your attention to detail. The full ethics statement has been included in the 'Methods' section of the manuscript. This section specifies the name of the IRB or ethics committee that approved the study, along with details regarding informed written consent.

***Copy of changes***

4. **Comment:** We note you have included a table to which you do not refer in the text of your manuscript. Please ensure that you refer to Table 1-5 in your text; if accepted, production will need this reference to link the reader to the Table.

**Response:** Thank you for your valuable feedback regarding the references to the tables in our manuscript. We have now included bold references to Tables 1-5 within the text of the manuscript to help readers easily locate them. This enhancement should improve the understanding of the data presented.

5. **Comment:** Please review your reference list to ensure that it is complete and correct

**Response:** Thank you for your feedback regarding the reference list. We have reviewed the reference list and removed reference 18 to ensure completeness and accuracy.

**Reviewers comment**

**Reviewer #1**

Thank you for your valuable feedback on our manuscript. We appreciate the time and effort you have dedicated to reviewing our work. Your insights are instrumental in enhancing the quality of the paper.

1. **Comment**: Title: Please, remove "PLOSE ONE".

**Response:** Thank you for your feedback regarding the title. We have removed "PLOS ONE" as requested.

2. **Comment:** Abstract-Conclusion: the main factors and relations should be enhaced in the conclusions.

**Response:** Thank you for your valuable feedback regarding the abstract and conclusion sections. We appreciate your suggestion to enhance the main factors and relations in the conclusions**.** The main factors and relationships in the conclusions have been enhanced to provide clearer insights.

***Copy of changes***

3. **Comment:** Method: The questionnaire is not clear for the reader, you only mention the number of items. To have an idea of the main questions, please add the questionnaire as Supplementary File. Did you use validated scales? Cite them with original authors. It is specially interesting to show the questions for protection motivation theory sub constructs. Please mention the possible bias that derived from a face-to-face interview. The Operational Definitions should be ordered in bullet points.

**Response:** Thank you for your valuable feedback regarding the clarity of the questionnaire. We appreciate your suggestions to enhance the manuscript. The questionnaire has been included as a Supplementary File to clarify the items used. Additionally, the validated scales, along with their original authors, have been cited. The potential biases arising from face-to-face interviews have also been addressed. The Operational Definitions have been reformatted into bullet points for improved clarity.

4. **Comment:** Result: Correct the typo in line174, 186, 196, 207, 230.

Subsection: line211 Screening attendance and associated factors. I recommend to increase the narrative description of the factors which decreased screening attendance: "Screening attendance decreased by 81% among women who had a higher response cost ([AOR] 0.19; [95% CI 0.08 to 0.50]) continue this exposition....

**Response:** Thank you for your valuable feedback on our manuscript. The typos noted on lines 174, 186, 196, 207, and 230 have been corrected. In the subsection on Screening Attendance, the narrative discussing the factors contributing to decreased screening attendance has been expanded for greater detail.

***Copy of changes***

5. **Comment:** Discussion: There are much paragraphs which start with "In this study..." "the findings" or similar. Please, vary these introductions to ideas. Ideas for improving the screening rates. I recommend to introduce a final paragraph with ideas, suggestions or examples of screening programs to try to solve the aforementioned problematic factors (national screening program, health education and knowledge, response costs, self-efficacy in intervention programs,

**Response:** Thank you for your valuable feedback regarding the repetitive phrasing in the manuscript. We appreciate your suggestion to vary the introductions to ideas, as well as your recommendation to include a final paragraph with suggestions for improving screening rates.

We have made an effort to vary the introductory phrases of paragraphs to avoid repetition. Additionally, we have included a final paragraph that suggests ideas for improving screening rates

***Copy of changes***

6. **Comment:** References: REf.18 is incorrectly cited in the list.

**Response:** Thank you for bringing the citation error in Reference 18 to our attention. We appreciate your careful review. We have removed the reference since the data is sourced directly from the Gedeo Zonal Health Department, and the source has been described within the manuscript itself.

**Reviewer #2**

1. **Comment:** This study was conducted on breast or cervical cancer screening and its predictors among Ethiopian women, the results of which can be useful for similar communities. The study has a good writing quality, I congratulate the authors for conducting the study, it is hoped that a similar studies will be conducted by screening other related factors. Some references are missing, they should be corrected.

**Response:** Thank you for your thoughtful feedback on our study. We sincerely appreciate your kind words about the writing quality and your recognition of the potential impact of our findings on similar communities. We acknowledge your comment regarding the missing references and have corrected this in the revised manuscript.

**Reviewer #3**

1. **Comment:** line 94 in method section: please mention the number of the women of 30-49 years age living in Gedeo zone.

**Response:** Thank you for your feedback. Unfortunately, the specific number of women aged 30-49 living in the Gedeo zone was not available in the data sources. I appreciate your understanding.

2. **Comment:** please include the formula used for calculating sample size.

**Response:** We acknowledge your recommendation to include the formula used for calculating sample size in the manuscript. We have included the formula used for calculating the sample size

***Copy of changes***

3. **Comment:** the sampling technique isn't clear for me; did you include the attendee of the selected health centers in your study? or the household in catchment Keeble ? or from both?. Also' you mention that "from each health center 30% of catchment Keeble was selected", why you specify 30%?

**Response:** Thank you for your insightful questions regarding the sampling technique used in our study. To clarify, our sampling method included participants from households within the catchment Kebeles. Regarding the selection of 30% catchment Kebele from each health center, this percentage would provide a representative perspective of the Kebeles.

***Copy of changes***

4. **Comment:** Add future clear parctical implication of the study's results as recoomendations at the conclusion section.

**Response:** Thank you for your valuable feedback. In the revised manuscript, we have added a section outlining clear practical implications of our results, including recommendations for health policy makers and health professional

***Copy of changes***

5. **Comment:** gramatical revision and paraphrasing is required

**Response:** We appreciate your insights and have made the necessary corrections to improve clarity and readability throughout the document.
